# Herbicide Resistance Management: Recent Developments and Trends

**DOI:** 10.3390/plants8060161

**Published:** 2019-06-08

**Authors:** Hugh J. Beckie, Michael B. Ashworth, Ken C. Flower

**Affiliations:** Australian Herbicide Resistance Initiative (AHRI), School of Agriculture and Environment, The University of Western Australia, Crawley, WA 6009, Australia; mike.ashworth@uwa.edu.au (M.B.A.); ken.flower@uwa.edu.au (K.C.F.)

**Keywords:** best management practices, crop competition, herbicide resistance, integrated weed management, precision weed management, site-specific weed management

## Abstract

This review covers recent developments and trends in herbicide-resistant (HR) weed management in agronomic field crops. In countries where input-intensive agriculture is practiced, these developments and trends over the past decade include renewed efforts by the agrichemical industry in herbicide discovery, cultivation of crops with combined (stacked) HR traits, increasing reliance on preemergence vs. postemergence herbicides, breeding for weed-competitive crop cultivars, expansion of harvest weed seed control practices, and advances in site-specific or precision weed management. The unifying framework or strategy underlying these developments and trends is mitigation of viable weed seeds into the soil seed bank and maintaining low weed seed banks to minimize population proliferation, evolution of resistance to additional herbicidal sites of action, and spread. A key question going forward is: how much weed control is enough to consistently achieve the goal of low weed seed banks? The vision for future HR weed management programs must be sustained crop production and profitability with reduced herbicide (particularly glyphosate) dependency.

## 1. Introduction

Over the past decade, the most significant change in strategy in herbicide-resistant (HR) weed management globally has been the increased focus on reducing the weed seed bank and maintaining low seed bank levels by whatever means possible. During the 1960s to 1990s, much research was devoted to determining the effect of weed density and time of emergence relative to the crop on crop revenue (yield by price) loss. These results were used to develop models or decision-support systems for herbicide application. Those initial weed economic threshold models with a single crop season focus became more sophisticated by considering longer time periods and weed seed bank implications, i.e., economic optimum thresholds or injury levels [1,2,3]. Today, the threshold concept, while still used in entomology and pathology, is no longer recommended by weed scientists or practitioners. The reality for most growers today is that the weed seed banks in their fields are likely resistant to one or more herbicide site(s) of action (SOA). A zero tolerance policy (‘take no prisoners’) is now being advocated [4,5]. However, it may not be agronomically and economically feasible or necessary to achieve this goal for all weed species and in all global cropping systems.

As detailed in the following sections, recent developments and trends related to HR weed management include renewed efforts in herbicide discovery by the agrichemical industry following a prolonged period from the late 1980s to present day when no new herbicide SOA was commercialized. In this intervening period, industry strategy has been to use existing chemistry for new, expanded uses by introducing single or combined (stacked) HR traits into our major agronomic crops, most notably soybean [*Glycine max* (L.) Merr.], maize (*Zea mays* L.), and cotton (*Gossypium hirsutum* L.) [6]. Although HR-trait stacking offers growers increased flexibility to manage HR weeds, the consensus of weed science academics is that this solution is not sustainable in the long-term with current practices and will inevitably lead to increased incidence of multiple-HR populations.

Another major trend related to herbicide usage is increasing prominence of preemergence (PRE) herbicides with soil-residual activity to fill the void left by postemergence (POST) products, such as acetyl-CoA carboxylase (ACCase) or acetolactate synthase (ALS) inhibitors, due to loss in efficacy because of widespread evolved resistance. Therefore, the pendulum is now swinging in the other direction. In the 1970s and 1980s, PRE herbicide application requiring soil incorporation dropped off markedly in favour of POST herbicide application more suited to low soil disturbance no-tillage cropping systems [7]. 

Additionally, we review two non-herbicidal HR weed management developments—one already making a significant impact (harvest weed seed control, HWSC) and one potentially posed to do so in the near future (weed-competitive crop cultivars). Although research and development into site-specific weed management (SSWM) (or precision weed management) have been ongoing for over 20 years, the adoption of this technology in agronomic field crops is still very low despite the demonstrated economic benefits [8]. Nevertheless, the significant potential economic and environmental benefits of SSWM cannot be ignored. We outline a relatively simple initial approach to SSWM that may help to spur grower adoption in the next decade.

## 2. Herbicide Discovery: Renewed Efforts

Herbicides remain an essential component of integrated weed management (IWM) systems in conventional agriculture. During the 1950s to 1970s, a new herbicide SOA was frequently introduced into the marketplace (Table 1 [9]). This pace of introduction slowed considerably in the 1980s and came to a standstill by the end of the decade. Presently, a herbicide with a new SOA has not been commercialized in more than 30 years. 

A key reason was the widespread adoption of glyphosate-resistant (GR) crops in the mid-1990s and concomitant increased use of glyphosate herbicide (Roundup™). Patent expiration in 2000 led to cheaper generic products and a marked increase in glyphosate usage. Glyphosate usage now surpasses that of any other herbicide SOA [10,11]. In addition, consolidation of the agrichemical industry into a few key players still supporting herbicide discovery programs and increasingly restrictive regulations have contributed to the current situation [12]. Today, we have a perfect storm of increasingly widespread global occurrence of multiple-HR weed populations in the face of increasingly limited herbicide SOAs due to weed resistance, uncertain economic returns from investment in herbicide discovery and development, and herbicide regulatory requirements or restrictions.

Discovering, developing, registering, and commercializing a new herbicide is a risky and costly venture, approaching USD $300 million [13]. On average, over 160,000 candidate compounds are screened to successfully introduce a new herbicide, with a development time from discovery to registration exceeding 10 years [14]. Nevertheless, industry has increased its efforts in recent years to find new molecules that may be promising future herbicides [15,16]. 

One promising direction is the discovery and development of novel herbicides based on products that are by-products of microorganisms or extracts of plants [17]. Research with natural phytotoxins has demonstrated several potential novel herbicide target sites; these compounds may lead to discovery of new SOAs [18]. Additionally, herbicidal properties of many antimalarial drugs suggest a possible source of compounds having novel SOAs [19]. Agrichemical companies have also adapted their discovery process to increase the probability of finding a new herbicide SOA, including molecular and enzyme structural modelling and SOA studies combined with a highly automated screening process [13]. Today’s molecular and other tools allow much more targeted and informed screening through genomics and metabolomics [12]. Collectively, these new or adapted technologies and processes used in herbicide discovery offer some optimism for new herbicide SOA by 2025 [20]. In the meantime, reliance on old herbicides registered for new and expanded uses (described in the following section) will have to bridge the gap.

## 3. Herbicide-Resistant (HR) Crops: The Changing State of Trait Adoption 

Soybean in the United States (U.S) perhaps best illustrates the changing adoption of cultivars with different HR traits (Table 2). The planted area of soybean in the U.S. in 2018 was 36.3 million ha [21]. Since 2014, the level of adoption of HR soybean in the U.S. has remained constant at 94% [22]. However, that statistic masks the changes occurring in adoption of different HR traits [23]. 

The dominant trait is still GR. The original Roundup Ready (RR) soybean went off patent in 2015. In 2009, RR2 soybean were introduced into the market. Although Bayer is transitioning to the RR2 Xtend (dicamba resistance) platform, many seed companies still sell RR2 cultivars. Currently, cultivars with the sole RR trait account for 30% of the U.S. market. The GR cultivars with the Xtend trait are expected to reach close to 50% of market share in 2019. This high adoption rate of the Xtend trait in just three years reflects its importance to growers in managing GR and other HR weeds. However, the dicamba herbicides (e.g., XtendiMax™) registered for use in these soybean cultivars are classified as a ‘restricted use pesticide’ by the U.S. Environmental Protection Agency. Therefore, they can only be sold to, and used by certified applicators in an effort to reduce potential injury to sensitive cultivars or crops due to off-target movement.

The LibertyLink™ (LL) soybean system, introduced in 2009, confers resistance to glufosinate (Liberty™) herbicide. In the merger with Monsanto, the technology was divested by Bayer to BASF (Credenz™ soybean platform). As with the RR2 Xtend soybean system, the LL system has been gaining market share over the past five years (currently about 20%) due to the need to manage the increasing incidence of GR weeds such as *Amaranthus* spp. This increased market share is also the result of lower seed prices in recent years and the availability of generic glufosinate herbicide products. Soybean cultivars with the combined RR+LL and RR+LL+dicamba (Xtend flex) traits are also available.

Going forward, GT27 soybean, developed by MS Technologies, Bayer and Mertec LLC, confer tolerance to glyphosate and isoxaflutole, an hydroxyphenyl-pyruvate-dioxygenase (HPPD)-inhibiting herbicide [23]. BASF introduced LL GT27, which will offer tolerance to three SOAs: glyphosate, glufosinate, and PRE-applied isoxaflutole. Another three-way stacked soybean system to be released is Enlist E3 developed by Dow AgroSciences and MS Technologies (now Corteva Agriscience), tolerant to glyphosate, glufosinate, and 2,4-D. 

A company’s decision to develop and introduce an HR trait for a particular crop depends primarily on the prospect for regulatory approval, market size (i.e., potential revenue), future market access, and grower demand (e.g., weed control needs). For example, frequent outcrossing between rice and related weed, red rice (*Oryza sativa* L.) was a consideration impacting the introduction of transgenic cultivars. Crops such as canola that are grown on a relatively small area globally are of lesser priority for investment in new HR-trait cultivars. Monsanto abandoned plans to commercialize GR wheat (*Triticum aestivum* L.) in 2004 largely because of lack of market acceptance of transgenic cultivars. Therefore, soybean, maize and cotton will continue to be the tier-1 level for investment, development, and introduction of stacked-HR trait cultivars.

With the rapid transition towards soybean and other crop cultivars with stacked (two- and three-way) HR traits, it will become more challenging for growers to choose the right system or systems to control HR and non-HR weeds on their farm. Comprehensive training for growers in proper stewardship practices of these technologies will be critical to optimizing and prolonging their benefits and minimizing risks as they are repeatedly and widely deployed across millions of hectares of cropland annually. Seed retailers and agronomists also require professional development training in this area as they often advise and influence a grower’s decision regarding herbicide options best tailored to their specific weed problems.

## 4. Increasing Prominence of Preemergence Herbicides

Over the past decade, there has been increasing reliance on preplant or PRE herbicides with short to long soil-residual activity to manage weed populations resistant to glyphosate and many POST herbicides such as ACCase or ALS inhibitors. For example, the use of preplant and PRE herbicides in U.S. soybean from 2000 to 2015 increased from 25 to 70% of crop area, largely in response to GR weeds [24]. The PRE herbicide SOAs commonly used in field crops include microtubule assembly inhibitors (dinitroanilines such as trifluralin), photosystem-II inhibitors (e.g., metribuzin), protoporphyrinogen oxidase (PPO) inhibitors (e.g., saflufenacil, flumioxazin, sulfentrazone), fat synthesis inhibitors (e.g., prosulfocarb, triallate), and very long chain fatty acid (VLCFA) inhibitors (e.g., pyroxasulfone [25]). 

Ideally, PRE herbicides will control weed cohorts very early in the growing season, thereby lessening potential crop yield loss due to weed competition and the selection pressure for resistance evolution from any in-crop (vegetative stage) or pre-harvest herbicide treatments that may still be required for acceptable season-long weed control. A PRE herbicide application is especially important for wide-row crops (e.g., maize, soybean, cotton) [26] or pulse crops (e.g., chickpea, *Cicer arientum* L.; lentil, *Lens culinaris* Medik.) with slow early-season growth and development or slow canopy closure. To proactively or reactively manage HR weeds in a growing season, the recommended herbicide treatment program should ideally comprise multiple SOAs applied as needed during one or more application windows (pre-seeding, in-crop, post-harvest) in mixtures or in sequence as required [27,28]. 

One case study that exemplifies this trend towards PRE herbicide usage is annual ryegrass (*Lolium rigidum* Gaud.) control in wheat in Australia. Traditionally, it was advised that growers should delay sowing in fields with high weed densities to maximize weed control before sowing and reduce the seed bank by the use of glyphosate or paraquat prior to sowing [29]. Today, however, wheat is often dry-seeded in a no-till system in April or early May to optimize yield potential. Common PRE herbicide treatments (soil incorporated by the seeding operation) in wheat include trifluralin, prosulfocarb + *S*-metolachlor, triallate, or pyroxasulfone. Growers are advised to integrate PRE herbicide application with multiple, effective cultural practices that optimize herbicide efficacy and crop suppression of weeds. With traditional delayed seeding to facilitate preplant weed control, weeds that establish in these late-sown winter crops can be more competitive due to the reduced crop growth rate associated with reduced soil and air temperatures. With the availability of highly-effective PRE herbicides that control initial weed cohorts, early seeding may be the best strategy to reduce weed seed production, as later germinating cohorts are suppressed by a larger crop canopy [30]. The effective control of summer annual weed species plus machinery innovations in no-tillage seeding now allow growers to efficiently establish crops earlier and make better use of available water resulting in increased yields [31,32].

To optimize the performance of these soil-applied herbicides, growers need to be aware of the multiple factors influencing their efficacy, including soil temperature; soil moisture; crop residue type, abundance and distribution; and degree of soil disturbance by tillage (ranging from minimal by disc openers to complete inversion by moldboard plow) and its impact on the distribution of soil organic carbon (organic matter) and texture (e.g., clay fraction) in the soil profile. A basic knowledge of the physical and chemical properties of each herbicide is needed to understand how it will likely behave in the soil environment of a grower’s field. Because these herbicides may be applied a few days before seeding or at the time of seeding (soil incorporation by sowing), the type of seeding equipment and operational parameters can affect PRE herbicide spatial (horizontal and vertical) distribution, which, in turn, is influenced by water distribution and abundance in the soil profile. When PRE herbicide application occurs in dry soil, research is needed to better understand the interaction between time of PRE herbicide application and time of rainfall (or time of seeding) in terms of crop injury and weed control efficacy. Additionally, more research is needed on agronomic practices that optimize the effectiveness and utility of PRE herbicides in conservation-tillage systems, such as crop seeding rate, row spacing, and fertilizer placement and timing.

## 5. Plant Breeders Consider Weed Competitiveness

Crop competitiveness against weeds is an important pillar of IWM and therefore HR weed management [33,34]. Weed scientists have long lamented that crop breeders have neglected to consider weed competitiveness in their breeding programs. Crop variety guides typically include yield, maturity, quality, and disease tolerance ratings, but provide no information on the degree of weed competitiveness. In the weed science community, breeding and selecting weed-suppressive crop genotypes have been a high priority for many years [35,36]. 

**Figure 1:**
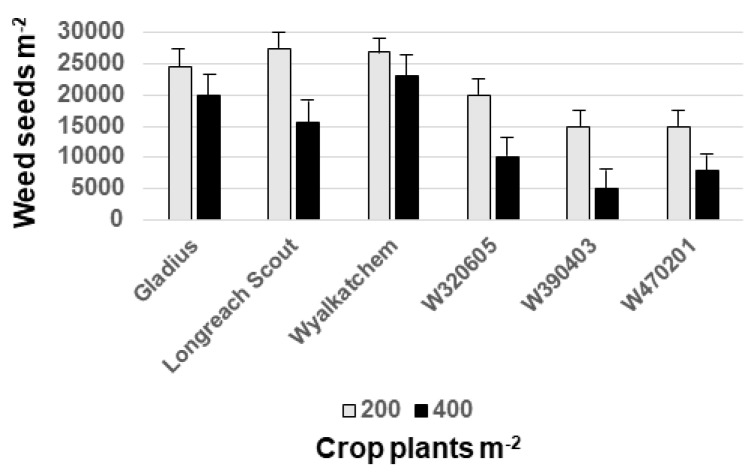
Weed seed production in field plots of wheat lines W320605, W390403 and W470201 with enhanced weed competitiveness compared with three commercial wheat cultivars (adapted from Rebetzke [37]).

With the continual increase in incidence and complexity of herbicide resistance in weed populations and declining availability of effective herbicide tools, plant breeders are now researching and developing germplasm with enhanced weed competitiveness (Figure 1; [37]). Research has identified both above- and below-ground traits that confer enhanced weed competitiveness. These traits include increased plant height, greater early vigour, and resource-competitive root systems. Early-season vigour is enabled by alternative dwarfing genes that do not reduce coleoptile length, which is a metric of weed competitiveness. Early ground cover (canopy closure) has been shown to be strongly correlated with the level of weed suppression. Germplasm that expresses weed-competitiveness traits are being developed in high-yielding genetic backgrounds. Weed-suppressive cereal (e.g., rice, wheat, barley (*Hordeum vulgare* L.)) cultivars will become increasingly available to growers over the next decade [37,38].

In future crop variety guides, we envision the inclusion of weed-competiveness ratings, with data collected annually from variety trial programs. Field phenomics/phenotyping uses non-destructive assessments (e.g., normalized difference vegetative index, NDVI) of crop growth vigour, such as percentage ground cover, canopy closure, or leaf area index. A first step, however, is validation of such assessments through controlled environment experiments on relative growth rates as well as small-plot experiments (weedy and weed-free conditions) measuring key parameters correlated with crop competitiveness [39]. Furthermore, evaluation of the weed-competitiveness rating system in the field should be performed using agronomic practices frequently used by growers. In the future, integrating agronomic practices such as fertilizer timing and placement, crop seeding rate and crop row spacing with weed-competitive cultivars will enhance the ability of the crop to suppress weeds and the opportunity for the grower to reduce their dependency on herbicide inputs. 

## 6. Harvest Weed Seed Control (HWSC) Gaining Momentum Globally

**Figure 2:**
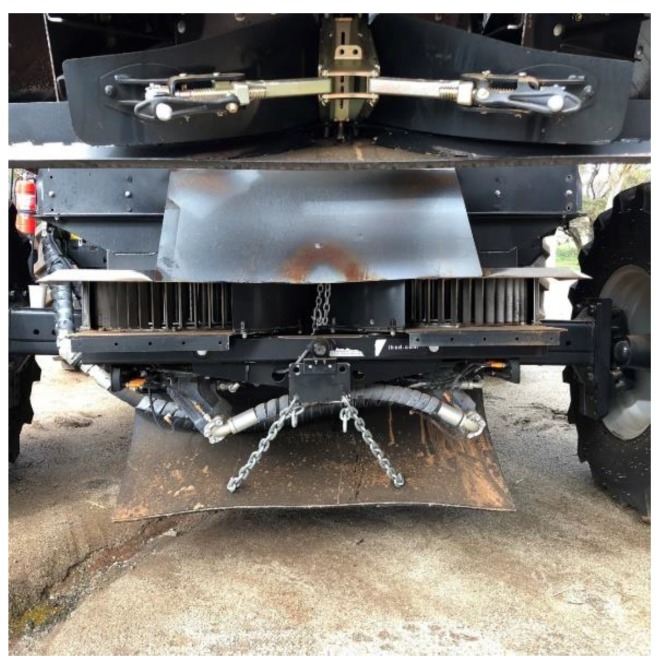
Mechanical seed destruction unit (iHSD) mounted in a commercial combine harvester.

HWSC is now an established, widely adopted weed management tool used by Australian grain growers following extensive research, development, and extension efforts. Various HWSC practices can greatly reduce the viability of weed seeds in the chaff fraction emitted by a combine harvester. These HWSC practices include the following (reviewed in Walsh et al. [40]): (1) narrow-windrow burning; (2) chaff cart towed behind the combine harvester; (3) bale-direct system (baler towed behind the combine harvester); (4) chaff-lining (chaff funneled into a narrow band behind the combine harvester); (5) chaff tram-lining (chaff directed onto the combine harvester wheel tracks; and (6) weed seed destruction (e.g., integrated HSD [41]; Figure 2). The adoption of narrow-windrow burning is significantly greater than that of the other HWSC practices [42]. Use of chaff carts, or to a lesser extent balers towed behind the combine harvester, is favoured by growers with livestock. However, the low cost of chaff-lining as well as the falling cost and improved engineering of weed seed destruction systems over time will likely increase grower adoption of these systems globally in the future.

Weed species most amenable to HWSC practices are those that retain their seeds in sufficient quantities until harvest (i.e., limited seed shatter) at a plant height (ca. 15 cm or higher) suitable for collection by the combine harvester. In Australia, two of the most problematic weeds, annual ryegrass and wild radish (*Raphanus raphanistrum* L.) meet both criteria well. In the last five years, substantial research and development efforts have been conducted in Canada and the U.S. to evaluate the potential of HWSC for key troublesome weed species, such as wild oat (*Avena fatua* L.) and cleavers (*Galium* spp.) in the Great Plains [43,44,45], and Palmer amaranth (*Amaranthus palmeri* S. Watson) and barnyardgrass (*Echinochloa* spp.) in the Midwest or southern U.S. [46]. Overall, results to date are encouraging. For example, only three of 20 weed species evaluated rated low potential for HWSC based on the criteria listed above (summarized in Walsh et al. [40]). In Europe, research is examining the potential for HWSC in management of HR blackgrass (*Alopecurus myosuroides* Huds.), despite substantial seed shatter before harvest, and other economically important weed species.

Ongoing field evaluation of different HWSC practices in different cropping systems across North America and elsewhere will facilitate grower awareness and future adoption of this important non-herbicidal weed management tool. An equally important consideration in these evaluations is the monitoring of weed species shifts over time as an inevitable consequence of the varying efficacy of HWSC practices on different weed species. In addition, weed populations may quickly adapt to recurrent HWSC practices in a field. Such adaptation may include earlier flowering and seed maturity, reduced seed retention on the plant, or plants that reproduce below harvest cutting height [47]. As with any recurrent weed management tool, whether herbicidal or not, we recommend close monitoring of changes in the phenology of weed populations as an adaptive response to reduce or evade exposure to this potential strong selection force.

Similar to herbicides, HWSC is not a stand-alone tool for weed management. Even on weed species well suited to seed capture or destruction at harvest such as annual ryegrass, weed control efficacy averages about 60% [48], not > 80% efficacy as typical for most herbicides. Therefore, HWSC is just one component of an IWM system, which ideally comprises combined (stacked) effective non-herbicidal practices that provide synergistic suppression of weed growth and fecundity.

## 7. Is Site-Specific Weed Management (SSWM) in Agronomic Field Crops Set to Take Off?

There is much hype surrounding ‘digital’ or ‘precision’ agriculture in relation to yield mapping, fertilizer application, etc. Currently, SSWM is mostly used in high-value, irrigated, traditionally labour-intensive horticultural crops [49]. To date, the rate of grower adoption of SSWM in larger-scale dryland agronomic field crops has been very slow despite the demonstrated economic benefits [8,50]. Are recent major corporate investments, such as John Deere acquisition of Blue River Technology or Monsanto (now Bayer) acquisition of The Climate Corporation (Climate FieldView™ software), a prelude to the take-off in development and adoption of SSWM in agronomic crops? 

**Figure 3:**
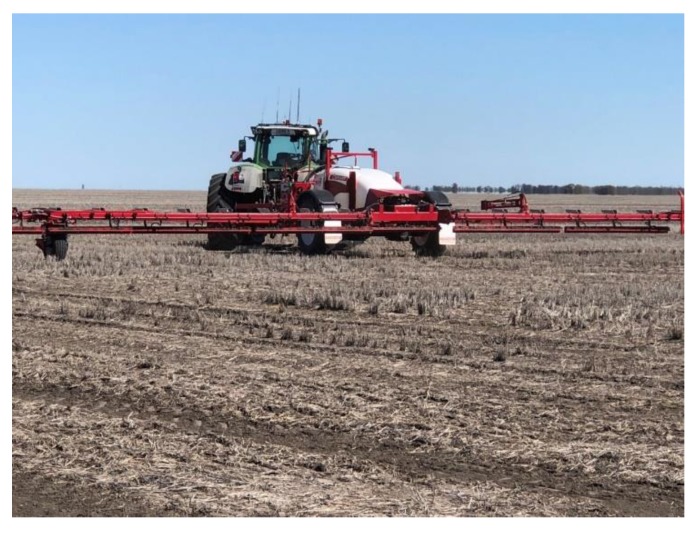
Autonomous site-specific weed control with a real-time weed detection and application sprayer in a fallow field.

Today, real-time weed control in fallow fields (‘green on brown’, e.g., WEED-It™ or WeedSeeker™ sprayers) is becoming more widespread in semi-arid to arid cropping regions (Figure 3). Herbicide savings of up to 90% have been reported [51]. Real-time weed detection/recognition and control in agronomic field crops (i.e., ‘green on green’) requires seamless integration and high performance of sensors, data processing, and actuation systems. Continuing technological advances in computer vision, robotics, machine learning, etc. are advancing this objective despite the many challenges that range from sensing weed vs. crop plants accurately (e.g., grass weeds in a cereal crop) while moving at speeds of up to 25 km h^-1^ to efficiently process and analyze large amounts of generated data [50,52]. Realizing the full potential utility of ‘big’ data for weed control is still conditional upon establishment of organizational, ethical and legal arrangements of data sharing [53]. 

**Figure 4:**
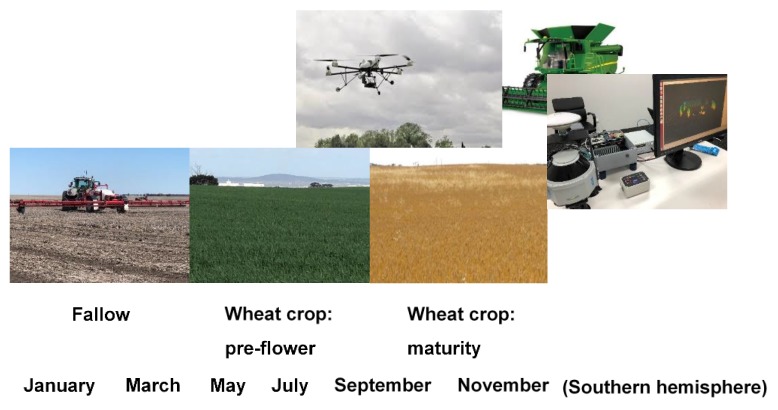
Growing season opportunities (semiarid southern hemisphere example) to map weed patches in wheat at pre-flower development stage using drone with mounted camera or near crop maturity using light detection and ranging (LiDAR) unit that could be mounted on a combine harvester; additionally, controlling weeds in fallow phase using a real-time weed detection sprayer.

Weeds often are not distributed evenly within fields, but tend to occur in patches of varying size, shape and density [54]. Weed patches are a visible manifestation of the underlying seed bank, which needs to be reduced over the period that seeds remain dormant or viable. Anecdotal observations of growers and agronomists suggest that weed populations are becoming less abundant and more patchy where HWSC has been frequently used in a field. Increasing occurrence of weed populations with a more patchy distribution as a result of recurrent HWSC is supported by modelling simulations. Recurrent HWSC in fields with multiple-HR weed populations was predicted to result in greater weed patchiness (spatial heterogeneity) across a field [55]. Under these circumstances, the cost-effectiveness of spraying the entire cropped field with a herbicide is questionable. In this case, a relatively simple initial approach to SSWM may be prudent (Figure 4). 

A first step in an elementary SSWM approach could be the creation of a weed map at crop maturity or time of harvest, using a global positioning system (GPS) unit mounted on a tractor or combine harvester. Such a map can be created manually (i.e, visually marked by the operator) or via mounted sensors with accompanying datalogger. Such sensors could include cameras, such as RGB (red, green, blue) or multispectral/color-infrared (3–7 bands around 100-nm width in the visible and infrared region); structure from motion; or light detection and ranging (LiDAR) system [50]. Such a monitoring system would be best suited to weed species with low propagule dispersal favouring patch stability or plants protruding above the crop canopy. Remote sensing in weed detection using unmanned aerial vehicles (UAVs) or drones can map weed patches in crops at early to late phenological stages and provide high spatial resolution images in a timely manner with relatively low operational costs [50,56,57]. Efficient data processing and geographic information system (GIS) mapping are required, with demonstrated accuracy (i.e., ground-truthing) of mapped weed patches. Until more technological advances are made in real-time weed control in agronomic crop fields, sequencing the operations of weed patch mapping and management is a less risky interim strategy. 

Seeding and spraying farm equipment are becoming increasingly capable of the variable rate application of inputs. Therefore, these weed patches could be targeted with greater crop seeding rates or highly effective herbicide treatments. Further research is needed to expand the use pattern of effective herbicides to enable label registration. As weed distributions would be mapped annually, growers would be able to continually adapt to changing weed distributions or newly evolving HR weed patches. With supporting research and development, this simple initial approach to SSWM may bridge the grower implementation disconnect that has characterized SSWM. The challenge for researchers is to work with growers and their agronomists and advisers to demonstrate (1) how existing technology can be adapted and used to easily and reliably map and manage weed patches in fields; and (2) sufficient return on investment. Grower experience and confidence gained using a relatively simple SSWM system may be an invaluable prerequisite for future adoption of more technologically-advanced real-time SSWM systems.

## 8. Conclusions and Future Outlook

The history of HR weed management globally shows that growers deal with the problem after it occurs, not before [58]. Furthermore, the extent of changes to their farming system depend upon the magnitude (distribution and abundance) and complexity (cross- and multiple-resistance patterns) of their HR weed populations in their fields. An increasing number of growers are now facing the prospect of changing crops or crop rotations to manage their HR weeds with remaining effective herbicides. For example, the percentage of farms in the United Kingdom adopting spring cropping as a means to manage HR grass weeds, such as blackgrass, increased from 32 to 81% from 2000 to 2016 [59]. Annual legume (pulse) crops are usually the weak link in the rotation because of their poor weed competitiveness combined with few registered herbicides or dependency on a few herbicide SOAs such as ALS inhibitors. These crop types are at greatest risk of declining planting area and production. The experience in Australia over the past decade is that effective PRE herbicides combined with agronomic practices to promote crop competition and minimize weed seed set or seed bank replenishment have generally resulted in sustained low weed seed bank levels of problematic weeds and profitable grain crop production. 

The vision for the future of HR weed management globally should center on reduced herbicide dependency, especially glyphosate. Non-herbicidal alternatives are often adopted to compensate for reduced herbicide efficacy due to increasing incidence of resistance, rather than as a partial replacement for herbicides [59]. The metric for successful IWM must be low weed seed banks concurrent with reduced herbicide use. What we are already witnessing is the merging of conventional and organic weed management strategies and tactics because of increasing incidence and impact of HR weed populations, increasing societal pressure on reducing pesticide use in food and feed production systems from perceived health and environmental perspectives, and increasing regulatory costs, requirements or restrictions surrounding pesticide registration and usage. Will herbicides be a ‘once in a century’ method of weed control due to widespread multiple-HR populations in many major cropping systems globally [60]? A key research question going forward is how much weed control is enough to consistently achieve the goal of low weed seed banks? This question implies reduced herbicide use, as measured by both treated area times the number of applications and herbicide loading (kg ha^-1^). Standardized methodology can be utilized to better assess the economic feasibility, impact, and consequences of reduced pesticide use [61]. To adequately address this question requires multi-site, medium-term (4–8 years) large-plot or landscape/field-level farming systems projects. Comparing key economic, agronomic, and environmental indices among farming systems, each with their unique crop sequence and combinations of weed management practices, can help identify more ecologically sustainable weed management systems. In addition, weed surveys and associated grower management questionnaires can help identify best management practices for maintaining low weed seed banks in reduced-herbicide farming systems.

## Figures and Tables

**Table 1:** Global introduction of herbicide site of action groups (adapted from Heap [9]; WSSA: Weed Science Society of America system used in the USA and Canada; HRAC: Herbicide Resistance Action Committee system used in all other countries except Australia, which has its own system).

Decade	Site of Action *	Example	WSSA	HRAC	Australia
1930s	Uncouplers (membrane disruption)	Dinoterb	24	M	Z
1940s	Synthetic auxins	2,4-D	4	O	I
	Auxin transport inhibitors	Diflufenzopyr	19	P	P
	Mitosis inhibitors	Propham	23	K2	E
1950s	Microtubule assembly inhibitors	Trifluralin	3	K1	D
	PS-II inhibitors	Atrazine	5	C1	C
	PS-II inhibitors (ureas and amides)	Chlorotoluron	7	C2	C
	Lipid inhibitors	Triallate	8	N	J
	Carotenoid biosynthesis inhibitors	Amitrole	11	F3	Q
	Nucleic acid inhibitors	MSMA	17	Z	Z
	PS-I electron diverters	Paraquat	22	D	L
1960s	PS-II inhibitors (nitriles)	Bromoxynil	6	C3	C
	PPO inhibitors	Oxyfluorfen	14	E	G
	VLCFA inhibitors	Metolachlor	15	K3	K
	Lipid inhibitors	Ethofumesate	16	N	J
	DHP synthase inhibitors	Asulam	18	I	R
	Cellulose inhibitors	Dichlobenil	20	L	I,O,Z
1970s	ACCase inhibitors	Diclofop	1	A	A
	ALS inhibitors	Chlorsulfuron	2	B	B
	Cell elongtion inhibitors	Difenzoquat	8	Z	Z
	EPSPS inhibitors	Glyphosate	9	G	M
	Glutamine synthase inhibitors	Glufosinate	10	H	N
	Carotenoid biosynthesis inhibitors (PDS)	Diflufenican	12	F1	F
	Antimicrotubule mitotic disrupters	Flamprop	25	Z	Z
1980s	DOXP inhibitors	Clomazone	13	F4	Q
	Cellulose inhibitors	Dichlobenil	21,26	L	I,O,Z
	HPPD inhibitors	Isoxaflutole	27	F2	H

* Abbreviations: ACCase: acetyl-CoA carboxylase; ALS: acetolactate synthase; DHP: dihydropteroate; DOXP: 4-deoxy-D-xylulose-5-phosphate synthase; EPSPS: 5-enolpyruvlshikimate-3-phosphate synthase; HPPD: hydroxyphenyl-pyruvate-dioxygenase; PDS: phytoene desaturase; PPO: protoporphyrinogen oxidase; PS: photosystem; VLCFA: very long chain fatty acid.

**Table 2:** Herbicide resistance (HR) traits in cultivars of major agronomic crops.

HR Trait	Soybean	Maize	Cotton	Rice	Canola	Wheat
ACCase inhibitor		X		X		X
ALS inhibitor		X		X	X	X
Triazine *					X	
Glyphosate	X	X	X		X	
Glufosinate	X	X	X		X	
Glyphosate+glufosinate	X	X	X			
Glyphosate+triazine*					X	
Glyphosate+dicamba	X		X			
Glyphosate+2,4-D+APP (ACCase)		X				
Glyphosate+isoxaflutole	X					
Glyphosate+glufosinate+dicamba	X		X			
Glyphosate+glufosinate+2,4-D	X		X			
Glyphosate+isoxaflutole+glufosinate	X					

* Australia only. Abbreviations: ACCase: acetyl-CoA carboxylase; ALS: acetolactate synthase; APP: aryloxyphenoxypropionate.
